# A feasibility study of ortho-positronium decays measurement with the J-PET scanner based on plastic scintillators

**DOI:** 10.1140/epjc/s10052-016-4294-3

**Published:** 2016-08-09

**Authors:** D. Kamińska, A. Gajos, E. Czerwiński, D. Alfs, T. Bednarski, P. Białas, C. Curceanu, K. Dulski, B. Głowacz, N. Gupta-Sharma, M. Gorgol, B. C. Hiesmayr, B. Jasińska, G. Korcyl, P. Kowalski, W. Krzemień, N. Krawczyk, E. Kubicz, M. Mohammed, Sz. Niedźwiecki, M. Pawlik-Niedźwiecka, L. Raczyński, Z. Rudy, M. Silarski, A. Wieczorek, W. Wiślicki, B. Zgardzińska, M. Zieliński, P. Moskal

**Affiliations:** 1 Faculty of Physics, Astronomy and Applied Computer Science, Jagiellonian University, S. Łojasiewicza 11, 30-348 Kraków, Poland; 2 INFN, Laboratori Nazionali di Frascati, CP 13, Via E. Fermi 40, 00044 Frascati, Italy; 3 Faculty of Physics, University of Vienna, Boltzmanngasse 5, 1090 Vienna, Austria; 4 Department of Nuclear Methods, Institute of Physics, Maria Curie-Sklodowska University, Pl. M. Curie-Sklodowskiej 1, 20-031 Lublin, Poland; 5 Świerk Computing Centre, National Centre for Nuclear Research, 05-400 Otwock-Świerk, Poland; 6 High Energy Department, National Centre for Nuclear Research, 05-400 Otwock-Świerk, Poland

## Abstract

We present a study of the application of the Jagiellonian positron emission tomograph (J-PET) for the registration of gamma quanta from decays of ortho-positronium (o-Ps). The J-PET is the first positron emission tomography scanner based on organic scintillators in contrast to all current PET scanners based on inorganic crystals. Monte Carlo simulations show that the J-PET as an axially symmetric and high acceptance scanner can be used as a multi-purpose detector well suited to pursue research including e.g. tests of discrete symmetries in decays of ortho-positronium in addition to the medical imaging. The gamma quanta originating from o-Ps decay interact in the plastic scintillators predominantly via the Compton effect, making the direct measurement of their energy impossible. Nevertheless, it is shown in this paper that the J-PET scanner will enable studies of the $\text{ o-Ps }\rightarrow 3\gamma$ decays with angular and energy resolution equal to $\sigma (\theta ) \approx {0.4^{\circ }}$ and $\sigma (E) \approx 4.1\,{\mathrm{keV}}$, respectively. An order of magnitude shorter decay time of signals from plastic scintillators with respect to the inorganic crystals results not only in better timing properties crucial for the reduction of physical and instrumental background, but also suppresses significantly the pile-ups, thus enabling compensation of the lower efficiency of the plastic scintillators by performing measurements with higher positron source activities.

## Introduction

The positron emission tomography (PET) is based on registration of two gamma quanta originating from a positron annihilation in matter. However, the $e^+ e^- \rightarrow 2 \gamma$ process is not the only possible route of positron annihilation. Electron and positron may annihilate also to a larger number of gamma quanta with lower probability, or form a bound state called positronium. In the ground state with angular momentum equal to zero positronium may be formed in the triplet state (with spin S = 1) referred to as ortho-positronium (o-Ps), or singlet state (S = 0) referred to as para-positronium (p-Ps). Positronium, being a bound-state built from electron and anti-electron bound by the central potential, is an eigenstate of both charge (C) and spatial parity (P) operators, as well as of their combination (CP). Therefore, it is well suited for the studies of these discrete symmetries in the leptonic sector. These symmetries may be studied by the measurement of the expectation values of various operators (odd with respect to the studied symmetry) constructed from the momenta of photons and the spin of the ortho-positronium [1]. Such studies are limited by the photon–photon interaction, however it was estimated that the vacuum polarisation effects may mimic the CP and CPT symmetries violation only at the level of 10$^{-9}$ [2], which is still by six orders of magnitude less than the presently best known experimental limits for CP and CPT violations in the positronium decays which are at the level of 0.3 % [3, 4]. Ortho-positronium is symmetric in space and spin and, therefore, as a system built from fermions it must be charge symmetry odd. Para-positronium, in turn, as anti-symmetric in spin and symmetric in space, must be charge symmetry even. C symmetry conservation implies that the ortho-positronium annihilate into odd number of gamma quanta, $3\gamma$ being the most probable, with lifetime 142 ns and para-positronium decays into even number of gamma quanta with lifetime 125 ps [5–8]. Such a huge difference in the life-times enables an efficient experimental disentangling of o-Ps from p-Ps decays.

**Fig. 1:**
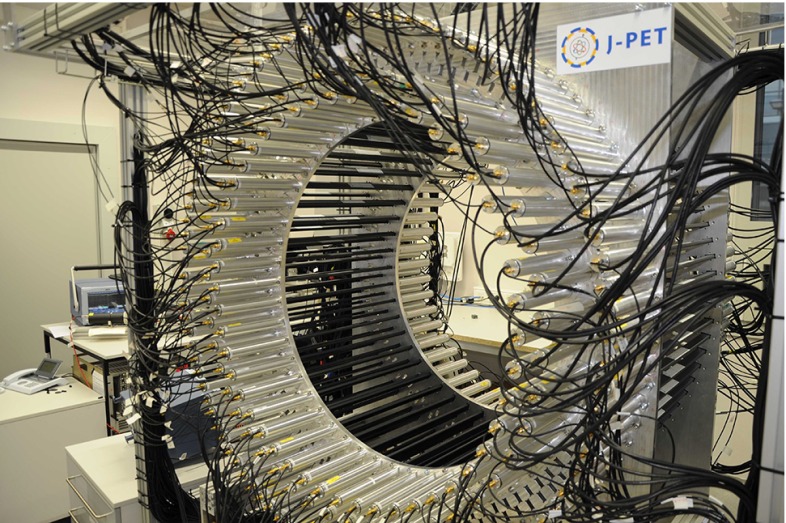
Photo of the Jagiellonian positron emission tomograph (J-PET). The J-PET detector is made of three cylindrical layers of EJ-230 plastic scintillator strips (*black*) with dimension of $7\times 19 \times 500 \text{ mm }^3$ and Hamamatsu R9800 vacuum tube photomultipliers (*grey*). The signals from photomultipliers are probed in the voltage domain at four thresholds with the timing accuracy of 30 ps [10] and the data acquisition is working in the trigger-less mode [11, 12]

With the recently constructed J-PET detector (see Fig. 1) we intend to study the $\text{ o-Ps }\rightarrow 3\gamma$ process in order to examine discrete symmetries and to test new medical imaging techniques based on the detection of three photons [9].

**Fig. 2:**
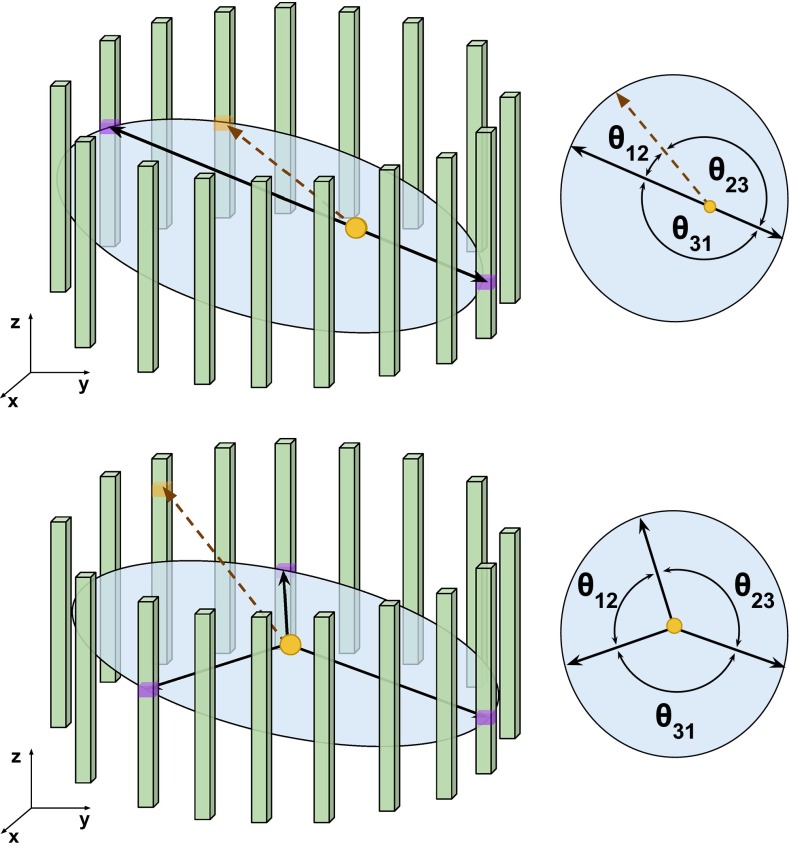
Schematic view of a single layer of the J-PET detector with two (*up*) or three (*down*) gamma quanta annihilation. In presently built geometry the first layer consists of 48 plastic scintillators (*green bars*). In this pictorial representation, for clarity, a smaller number of strips is shown. *Solid dark blue lines* indicate annihilation quanta and *dashed brown line* indicates de-excitation gamma quantum e.g. from the $^{22} \text{ Na } \rightarrow \ ^{22}\text{ Ne }^* + e^+ + \nu \rightarrow \ ^{22}\text{ Ne } + \gamma + e^+ + \nu$ decay chain. Due to the momentum conservation annihilation quanta are moving along the same line in the case of $e^+e^- \rightarrow 2\gamma$, while in the case of the $e^+e^- \rightarrow 3\gamma$ they are included in a single plane. The de-excitation photon (*dashed line*) is not correlated with the annihilation photons and is isotropically distributed with respect to the annihilation plane-of-response. Due to the fact that annihilation and de-excitation occur in a good approximation at the same place the photons from the $e^+e^- \rightarrow 2\gamma$ form a plane with the de-excitation photon

In the ortho-positronium decay the additional information carried by the 3rd $\gamma$ allows more precise annihilation point reconstruction. Schematic view of p-Ps and o-Ps annihilation is shown in Fig. 2.

Moreover, the observed yield of three gamma annihilation depends on material’s properties (see Sect. 2.1), therefore it may allow to gain some information not only about location but also about properties of tumors [13]. In fundamental physics, studies of the three gamma annihilation allows not only to test the discrete symmetry violation [14] but also enables searches of physics beyond the Standard Model: extra dimensions [15], dark matter [16] and a new light vector gauge boson [17]. Since a detailed physics program of J-PET and its motivation is described elsewhere in a dedicated article [1], here as an example we would like only to discuss briefly experimental approach to determining the expectation value of the odd operator for the CPT symmetry, whose violation has not been observed so far. As it was recently shown [18] the J-PET detector allows for a spin direction ($\mathbf {S}$) determination of o-Ps created in cylindrical target. Additionally as it is described in Sect. 5 the J-PET detector enables determination of the momentum vectors of gamma quanta originating from the $\text{ o-Ps }\rightarrow 3\gamma$ process. These properties allow for construction of the following operator odd under CPT transformation: $\mathbf {S}\cdot (\mathbf {k_1}\times \mathbf {k_2})$, where $\mathbf {k_1}$ and $\mathbf {k_2}$ denote momenta of the most and second most energetic quanta, respectively. The non-zero expectation value (indicating violation of CPT symmetry) would manifest itself as an asymmetry between numbers of events with spin direction pointing to opposite sides of the decay plane ($\mathbf {k_1}\times \mathbf {k_2}$). In this paper we focus on the feasibility study of the detection of o-Ps annihilation.

**Table 1:** Summary of major physical characteristics of beta-plus isotopes useful for PET imaging and positron annihilation lifetime spectroscopy (PALS) investigations. For isotopes that decay into excited states the properties of emitted gamma quanta are denoted. Data were adapted from [27]

Isotope	Half-life	$\beta ^+$ decay	$E_{\gamma }$ (MeV)	$E_{e^+}^{max}$ (MeV)	Excited nuclei lifetime
Isotopes for PALS and PET imaging					
$^{22}$Na	2.6 (years)	$^{22} \text{ Na } \rightarrow ^{22}\text{ Ne } + e^+ + \nu _e +\gamma$	1.27	0.546	3.63 (ps)
$^{68}$Ga	67.8 (min)	$^{68}\text{ Ga } \rightarrow ^{68}\text{ Zn } + e^+ + \nu _e +\gamma$	1.08	0.822	1.57 (ps)
$^{44}$Sc	4.0 (h)	$^{44}\text{ Sc } \rightarrow ^{44}\text{ Ca } + e^+ + \nu _e +\gamma$	1.16	1.474	2.61 (ps)
Isotopes for PET imaging					
$^{68}$Ga	67.8 (min)	$^{68}\text{ Ga } \rightarrow ^{68}\text{ Zn } + e^+ + \nu _e$	–	1.899	–
$^{11}$C	20.4 (min)	$^{11}\text{ C } \rightarrow ^{11}\text{ B }+ e^+ + \nu _e$	–	0.961	–
$^{13}$N	10.0 (min)	$^{13}\text{ N } \rightarrow ^{13}\text{ C }+ e^+ + \nu _e$	–	1.198	–
$^{15}$O	2.0 (min)	$^{15}\text{ O } \rightarrow ^{15}\text{ N }+ e^+ + \nu _e$	–	1.735	–
$^{18}$F	1.8 (h)	$^{18}\text{ F } \rightarrow ^{18}\text{ O } + e^+ + \nu _e$	–	0.634	–

The registration of three-gamma annihilation and conducting of the above mentioned research is possible by the J-PET detector whose novelty lies in application of plastic scintillators instead of crystals [19]. This solution allows to sample fast signals (5 ns) [10, 20–23] and build more extended geometries, in comparison to commercially used PET detectors [20]. In this paper we study the feasibility of the three gamma annihilation measurements using the J-PET detector. To this end we have developed Monte Carlo simulations accounting for:

(i) positron emission and thermalisation in the target material,
(ii) angular and energy distributions of gamma quanta originating from ortho-positronium annihilation,
(iii) Compton interactions of emitted gamma quanta in the detector built from plastic scintillators,
(iv) determination of gamma quanta hit-position and hit-time in the detector with experimentally determined resolutions,
(v) multiple scattering and accidental coincidences,
(vi) reconstruction of registered gamma quanta four-momenta,

and used four possible geometrical configurations of the J-PET detector.

Section 2 gives a general introduction of positron emission and interaction with matter together with the formation of positronium and the description of ortho-positronium annihilation into three gamma quanta. Possible detector geometries are summarized in Sect. 3. Properties of J-PET detector, comparison between simulated and experimental spectra and the method of background rejection are presented in Sect. 4. Section 5 contains the detector efficiency estimation as well as the energy and angular resolutions.

## Performance assessment: Monte Carlo simulations

The following paragraphs contain the description of Monte Carlo simulations of positrons emitted from $\beta ^+$ source ($^{22}$Na) that bind with electron and form positronium. Simulation takes into account the effects of finite positronium range and non-zero residual momentum of the annihilation positron-electron pair. Special emphasis is put on a proper description of available phase-space of photons from ortho-positronium annihilation and their further detection in the J-PET detector that consists of plastic scintillators.

### Positron source and positronium formation

Table 1 summarizes the important characteristics of the isotopes used for different types of imaging as well as in laboratory studies. Those isotopes decay through $\beta ^+$ transitions emitting a positron that travels through matter, scatters and slows down reaching thermal energies. Then it undergoes free annihilation or forms a positronium [24]. In water at $20~^{\circ }$C the positron has about $64~\%$ chance of undergoing free annihilation [25]. The positronium is produced mostly in the ground state forming para-positronium ($^1S_0$, p-Ps) or ortho-positronium ($^3S_0$, o-Ps) with probability of 25 and $75~\%$, respectively. The annihilation of those states is leading predominantly to an emission of two or three gamma quanta for p-Ps or o-Ps states, respectively. However, the interactions with matter can lead to inversion of the ortho-positronium spin or to the pick-off processes and, as a result, can affect the relative ratio of $3\gamma /2\gamma$ annihilation. The effective yield of annihilation into $3\gamma$ in most of non-metallic substances is of the order of $1~\%$, although in some cases, as for example fine powders of alkaline oxides, it can reach even 29 % as recently shown for the amberlite porous polymer XAD-4 (CAS 37380-42-0) [26].

**Fig. 3:**
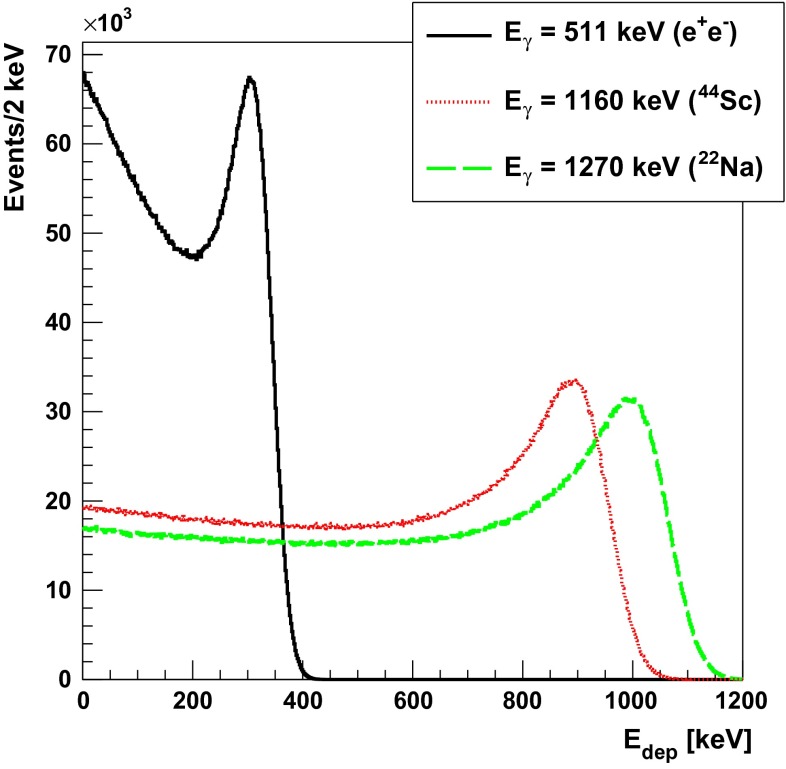
Simulated spectra of deposited energy in plastic scintillators for gamma quanta from $e^+ e^- \rightarrow 2 \gamma$ annihilation and for de-excitation gamma quanta originating from isotopes indicated in the legend. The spectra were simulated including the energy resolution of the J-PET detector [20] and were normalized to the same number of events

**Fig. 4:**
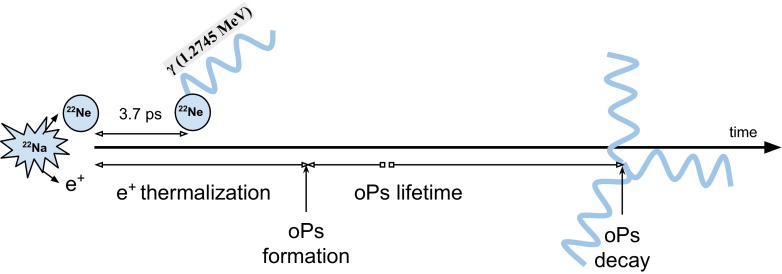
Scheme of sodium decay and formation of ortho-positronium

Some of the $\beta ^+$ emitters, e.g. $^{22}$Na or $^{44}$Sc, decay to daughter nucleus in excited states and emit prompt gamma with a well defined energy. In plastic scintillators gamma quanta interact mostly via the Compton scattering. Figure 3 shows the energy loss spectrum expected for the gamma quanta from the $e^+e^- \rightarrow 2 \gamma$ annihilation compared to the spectra expected from the de-excitation quanta from $^{22}$Na and $^{44}$Sc isotopes.

The results were obtained taking into account the experimental energy resolution of the J-PET detector [28]. The identification of de-excitation and annihilation photons is based on the energy loss and angular correlations. Using the energy loss criterion (e.g. $E_{dep} > 0.370$ MeV) we can uniquely identify de-excitation quantum from the $^{44}$Sc and $^{22}$Na decays with a selection efficiency of 0.66 and 0.70, respectively. The second selection method is, however, much more efficient. It will be based on the relation between the relative angles of the photons directions. The trilateration method allows reconstruction of an emission point [18] and the relative angles between the gamma quanta. After assigning the numbers to the photons such that the relative angles are arranged in the ascending order ($\theta _{12}< \theta _{23} < \theta _{31}$), in the case of the 2$\gamma$ annihilation (Fig. 2, left) the largest angle $\theta _{31}$ will be equal to 180 degrees and will correspond to the photons from the $e^+ e^- \rightarrow 2 \gamma$ process. Therefore, the de-excitation gamma quantum can be identified as photon number 2. This second selection method is independent of the energy loss criteria, and due to the high angular resolution of the J-PET tomograph (see Sect. 3), it will allow an identification with close to 100 % selection efficiency. In case of the 3$\gamma$ annihilation, photons originating from $\text{ o-Ps }\rightarrow 3\gamma$ process are emitted in a single plane. The gammas directions are not correlated with the de-excitation photon (see Fig. 2), so probability of their miss-identification with the de-excitation gamma quanta is at the level of few percent only, and for long lifetime of o-Ps (larger than few ns) it is negligible due to the large difference between the hit-times of annihilation and de-excitation photons which may be used as additional third criterion.

**Fig. 5:**
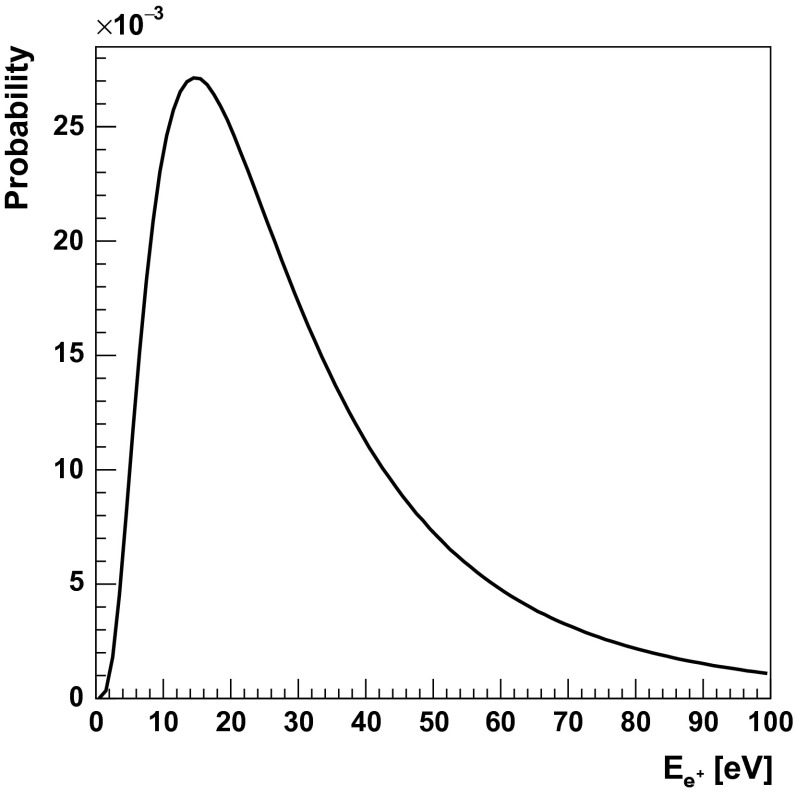
Simulated probability density function of positronium formation as a function of positron energy after thermalisation in the water. The distribution is adapted from reference [29]

**Fig. 6:**
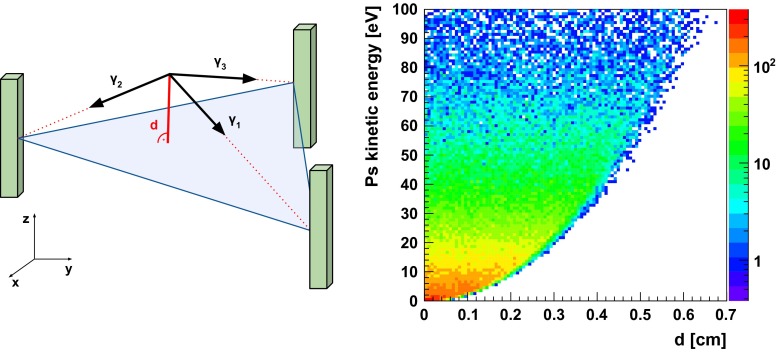
*Left* Scheme of the ortho-positronium annihilation into three gamma quanta $\gamma _i$ in the detector reference frame. Gamma quanta are not contained in a single plane due to non-zero kinetic energy of the ortho-positronium. In the experiment a plane of response can be determined from gamma quanta interaction position in the scintillators (*green bars*). The distance *d* between plane of response and annihilation vertex gives information about annihilation position uncertainty. *Right* Distribution of distance *d* as a function of kinetic energy of ortho-positronium. Taking into account resolution of the J-PET annihilation point reconstruction [18], the uncertainty caused by o-Ps’s boost is negligible

**Fig. 7:**
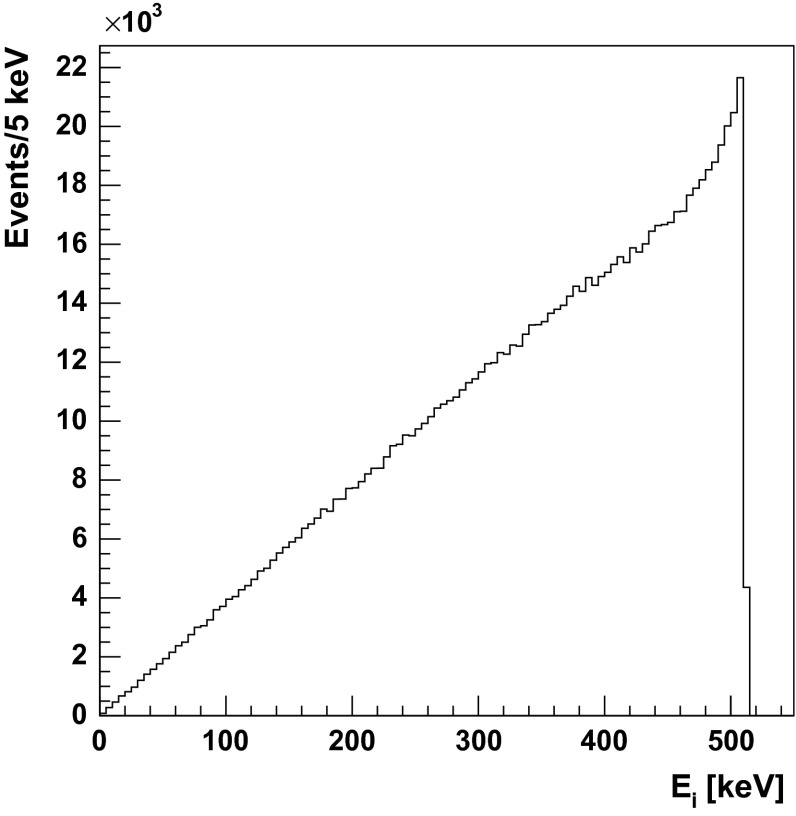
Energy spectrum of photons originating from three-photon annihilation of an electron and a positron

In further considerations we will focus on sodium isotope, which is commonly used as a source of positrons for various experiments and tests of detectors. Pictorial representation of the studied $\text{ o-Ps }\rightarrow 3\gamma$ process is shown in Fig. 4.

In the conducted simulations we took into account the description of positron properties after thermalisation. Its energy was simulated according to the distribution presented in Fig. 5 [29].

The distribution of the initial positron kinetic energy depends only on thermalisation processes. This distribution is taken into account in the transformation of gamma quanta four-momenta from the rest frame of ortho-positronium to the laboratory frame. In addition, the small distance traveled by positron in matter was taken into account. Positron range depends on material properties and can be generated from profiles known in the literature [30] provided by many simulation packages, such as GATE [31] or PeneloPET [32]. In this work the positron range distribution obtained by PeneloPET was adopted. Abovementioned effects introduce additional smearing of o-Ps annihilation position (see Fig. 6) and are included into performed simulations.

### $\text{ o-Ps }\rightarrow 3\gamma$ process

**Fig. 8:**
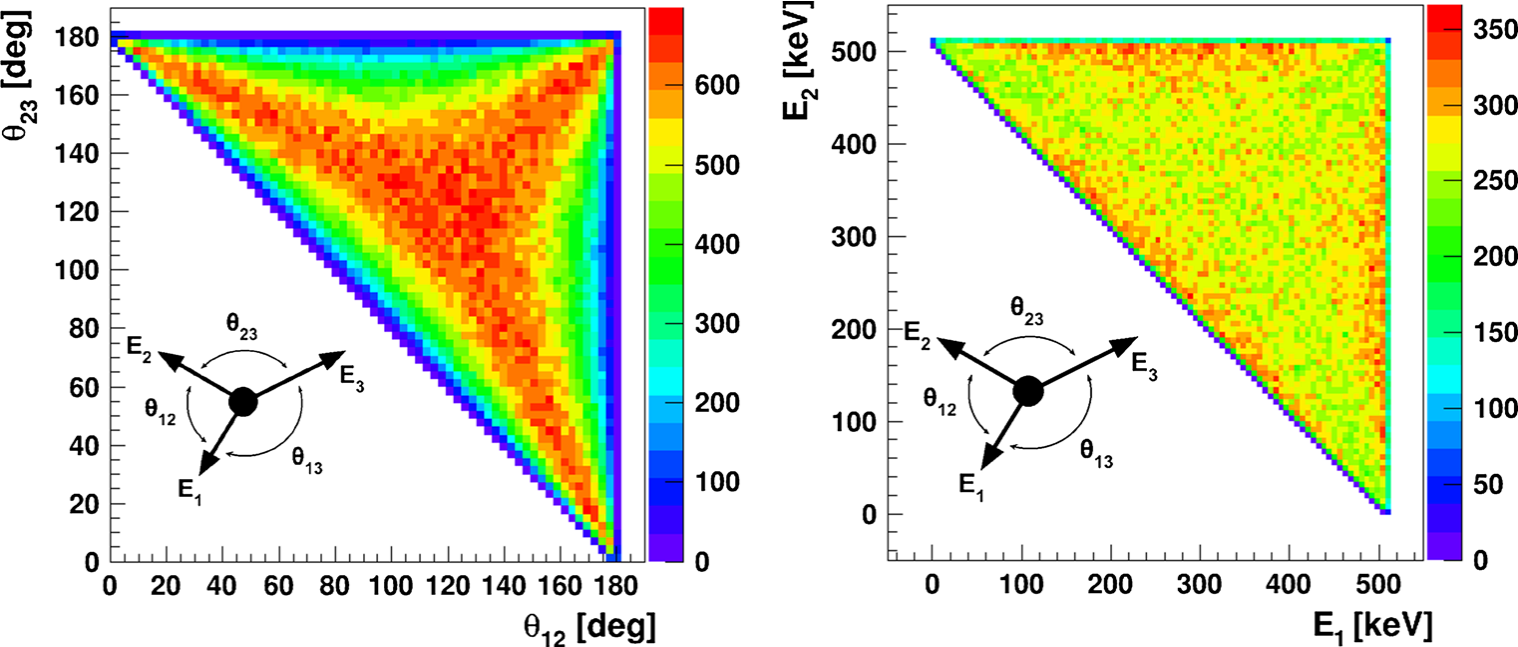
Distribution of angles (*left*) and Dalitz plot (*right*) of $\text{ o-Ps }\rightarrow 3\gamma$ annihilation. Boundaries are determined by kinematic constraints

Positronium is the lightest purely leptonic system, and it can annihilate only into gamma quanta. Those photons are coplanar in the Center of Mass (CM) frame due to the momentum conservation. The cross-section for annihilation with formation of photons having frequencies $\omega _i$ can be expressed as [33]:

1 $$\begin{aligned} \sigma _{3\gamma }&= \frac{4 e^6}{v m_e^2} \cdot \int _{0}^{m_e} \int _{m_e-\omega _{1}}^{m_e} \frac{\left( \omega _1 + \omega _2 - m_e \right) ^2}{\omega _{1}^2 \omega _{2}^2} d \omega _1 d \omega _2 \nonumber \\&= \frac{4 e^6}{v m_e^2} \cdot \frac{\pi ^2 - 9}{3} \end{aligned}$$

where $m_e$ is electron mass, *v* denotes electron-positron relative velocity, *e* is the elementary charge. In above formula the conservation of 4-momentum allows to eliminate one of the frequencies ($\omega _{3}$). Equation 1 results in the characteristic energy distribution of gamma quanta (see Figs. 7, 8).

**Fig. 9:**
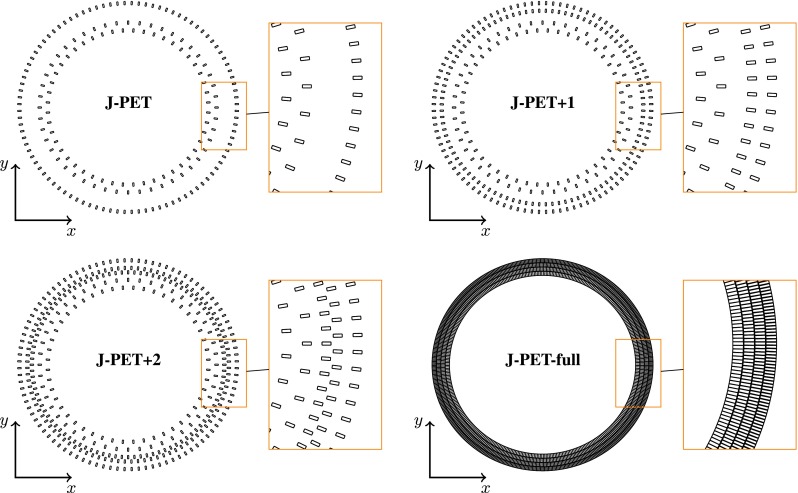
Transverse view of simulated geometries

**Table 2:** Details of simulated layers of the J-PET geometry. J-PET detector has been already built [1]. The mechanical construction for the next phases J-PET+1 and J-PET+2 is also prepared and the hardware upgrade is planned within the next 2 years

Layer number	Layer radius with respect to the center of scintillator (cm)	Number of scintillators in the layer	Angular displacement of $n_i$ scintillator
J-PET			
1	42.50	48	$n_i \times 7.5^{\circ }$
2	46.75	48	$n_i \times 7.5^{\circ } + 3.75^{\circ }$
3	57.50	96	$n_i \times 3.75^{\circ } + 1.875^{\circ }$
J-PET+2			
1	42.50	48	$n_i \times 7.5^{\circ }$
2	46.75	48	$n_i \times 7.5^{\circ } + 3.75^{\circ }$
3	50.90	96	$n_i \times 3.75^{\circ }$
4	53.30	96	$n_i \times 3.75^{\circ } + 1.875^{\circ }$
5	57.50	96	$n_i \times 3.75^{\circ } + 1.875^{\circ }$
J-PET+1			
1	42.50	48	$n_i \times 7.5^{\circ }$
2	46.75	48	$n_i \times 7.5^{\circ } + 3.75^{\circ }$
3	53.30	96	$n_i \times 3.75^{\circ }+1.875^{\circ }$
4	57.50	96	$n_i \times 3.75^{\circ }+1.875^{\circ }$
J-PET-full			
1	43.0	400	$n_i \times 0.9^{\circ }$
2	45.0	437	$n_i \times 0.82^{\circ }$
3	47.0	473	$n_i \times 0.76^{\circ }$
4	49.0	508	$n_i \times 0.71^{\circ }$

## Simulated geometries

A few possible detector geometries were simulated. They are referred to as J-PET, J-PET+1, J-PET+2 and J-PET-full:

J-PET corresponds to the already built detector [1] with 3 layers of scintillators (from in to out: 48 + 48 + 96 scintillators).
J-PET+1 the J-PET geometry extended by an additional layer filled by 96 scintillators.
J-PET+2 geometry assumes complete fulfillment of all available layers in the J-PET detector (48 + 48 + 96 + 96 + 96).
J-PET-full detector with fully coverage of four plastic scintillator layers.

The details of the simulated geometries are presented in Fig. 9 and Table 2. Comparison of the results for all above mentioned options shows the accuracy of $\text{ o-Ps }\rightarrow 3\gamma$ registration achievable at current J-PET setup and upgrades planned in the next two years, as well as for the J-PET-full detector. In all the cases simulations assume the usage of EJ-230 plastic scintillator strips (dimensions $1.9 \text{ cm } \times 0.7 \text{ cm } \times 50.0 \text{ cm }$), with the longest side of the scintillator arranged along the *z* axis.

## J-PET detector properties

The multipurpose detector (J-PET) constructed at the Jagiellonian University of which novelty lies in using large blocks of plastic scintillators instead of crystals as detectors of annihilation quanta, requires the usage of the time of signals, instead of their amplitude, and allows to obtain time resolution better than 100 ps [28].

### Determination of hit and time position at J-PET

**Fig. 10:**
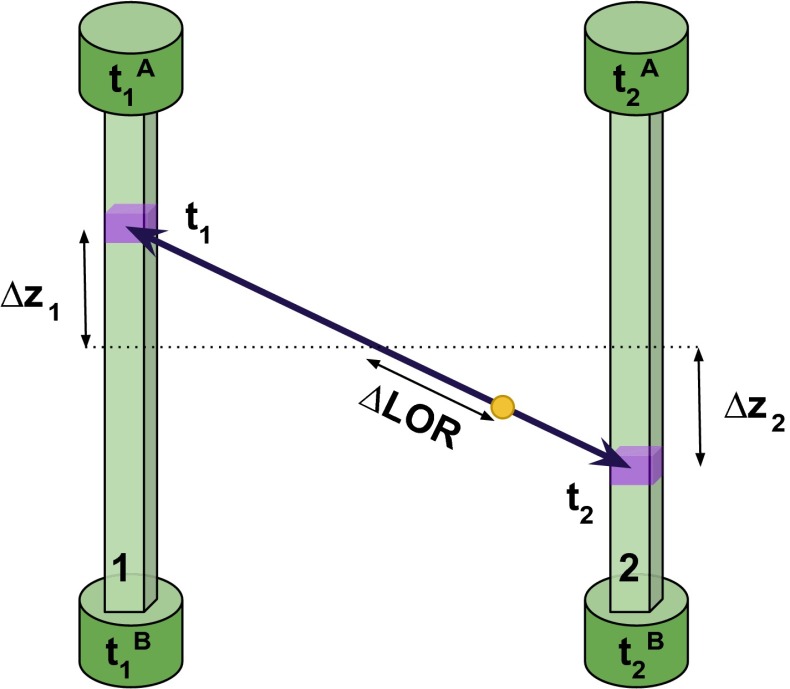
Registration of the signals arrival time on the two ends of a single scintillator ($t_1^A$, $t_1^B$ and $t_2^A$, $t_2^B$ for the first and second strip, respectively) allows to determine the distance from the scintillators centers ($\Delta z_{1,2}$) and times ($t_{1,2}$) when gamma quanta interacts with scintillators. Then the line of response can be determined as well as the displacement of the annihilation position from its center ($\Delta \text{ LOR }$)

Reconstruction of time and gamma quanta hit position in *i*th plastic scintillator can be based on the time values ($t_i^A$, $t_i^B$) of scintillation light registration in photomultipliers located at the ends of single plastic scintillator strip. Then the distance $(\Delta z_i)$ along the strip between its center and the hit position can be expressed as:

2 $$\begin{aligned} \Delta z_i = \frac{(t_i^A - t_i^B)\cdot v}{2}, \end{aligned}$$

where *v* is the light velocity in the plastic scintillator. Based on this information, in case of two-gamma quanta annihilation, the line of response (LOR) and the annihilation position along it can be determined (see Fig. 10).

In case of three-gamma annihilation, the registered gamma quanta are coplanar (o-Ps kinetic energy can be neglected, see Sect. 2.1) and the registered hit-points form the plane-of-response (POR) (see Fig. 2, right panel). In this case the annihilation position can be determined using the novel reconstruction based on trilateration method (see Sect. 5.1.2). The obtained energy and time resolution of registered gamma quanta were experimentally determined and within the range of deposited energy ($E_{dep}$) $\in (200,340) \text{ keV }$, are equal to [28]:

3 $$\begin{aligned}&\sigma ( T^0_{hit} ) \approx 80 \text{ ps } , \end{aligned}$$

4 $$\begin{aligned}&\frac{\sigma (E)}{E} = \frac{0.44}{\sqrt{ E \text{[MeV] } }}. \end{aligned}$$

For lower energies the time resolution can be expressed as a function of deposited energy ($E_{dep}$):

5 $$\begin{aligned} \sigma ( T_{hit} (E_{dep})) = \frac{ \sigma (T^0_{hit}) \text{[ps] } }{ \sqrt{\frac{E_{dep} \text{[keV] }}{270}} }. \end{aligned}$$

Considering the most challenging time reconstruction for gamma quanta with low energies (around 50 keV), one can see that the J-PET detector provides a precision on the level of two hundred picoseconds. In the commercial PET systems the events with an energy deposition lower than about 400 keV [36, 37] are discarded.

**Fig. 11:**
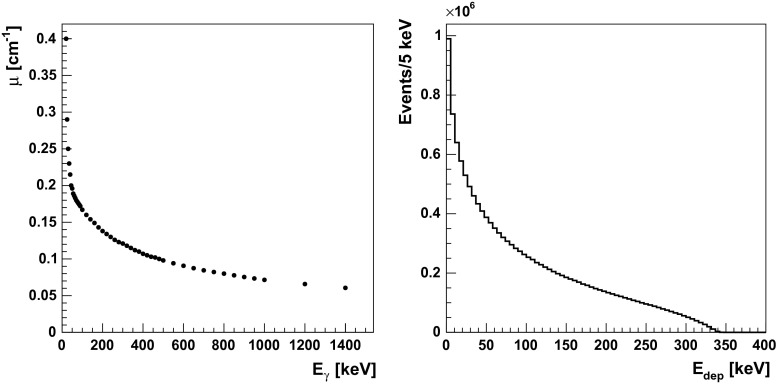
*Left* dependency of attenuation coefficient on incident gamma quanta energy. Data taken from [34]. *Right* distribution of energy deposited by gamma quanta in plastic scintillators originating from $\text{ o-Ps }\rightarrow 3\gamma$ annihilations. The shown spectrum is a convolution of the energy distribution of gamma quanta from the $\text{ o-Ps }\rightarrow 3\gamma$ decay (Fig. 7) and the Klein–Nishina distribution of kinetic energy of electrons acquired via Compton scattering [35]. Spectrum includes the absorption dependence on the energy (*left panel*) and the detector energy resolution

### Spectra of deposited energy

The probability of incident gamma quanta registration is a function of the attenuation coefficient $\mu$ and distance that gamma quantum travels through the material. In the simulations the attenuation coefficient was parametrized as a function of incident gamma quanta energy (see Fig. 11, left panel).

**Fig. 12:**
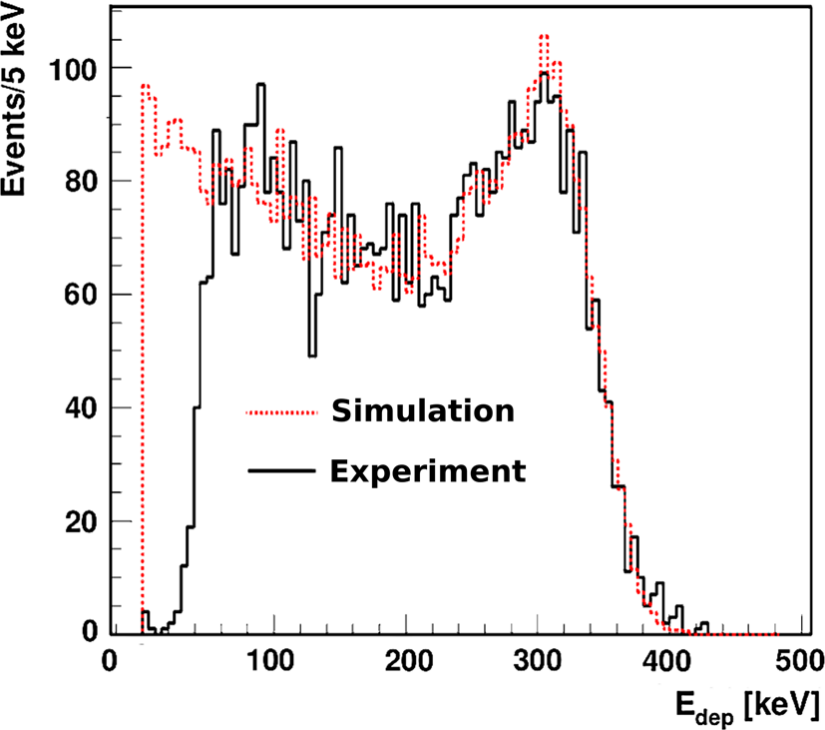
Spectra of simulated (*red*, *dashed line*) and measured energy (*solid*, *black line*) deposition by 511 keV gamma quanta in J-PET detector. The simulated spectrum was normalized to the experimental one, and simulations were performed taking into account the energy resolution (Eq. 4). The *left part* of the experimental spectrum was cut due to the triggering threshold applied in the experiment

Gamma quanta interact with plastic scintillators mainly via Compton effect and the characteristic spectra of deposited energy are described by Klein-Nishina formula [35, 38]. The distribution for 511 keV incident gamma quantum is shown in Fig. 12. Energy of single gamma quanta from ortho-positronium annihilation is within [0, 511] keV energy range and the spectrum of deposited energy via Compton effect for the corresponding energy range is presented in Fig. 11 (right panel).

**Fig. 13:**
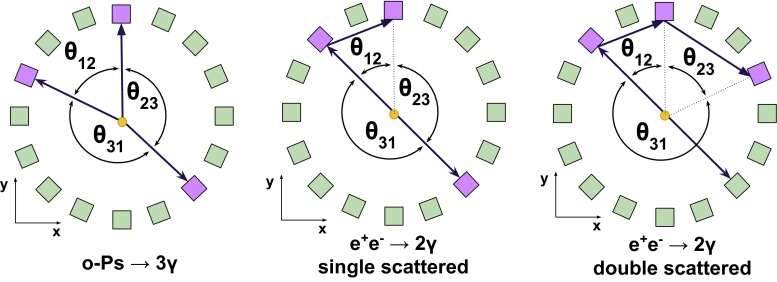
Pictorial illustration of the possible response of the detector to $\text{ o-Ps }\rightarrow 3\gamma$ and $e^+e^-$ annihilation into $2\gamma$. Arranged circularly *squares* represents scintillator strips—*purple* and *green* colors indicate strips where the gamma quanta were or were not registered, respectively. The *arrows* represents gamma quanta occurring in the events, while *dotted lines* indicate naively reconstructed gamma quanta. Examples of primary and secondary scatterings are depicted

### Background rejection

Direct annihilation of positron with electron, as well as intrinsic annihilation of para-positronium, are both characterized by short times of $\sim$400 and $\sim$125 ps, respectively. For comparison, an ortho-positronium lifetime in vacuum amounts to about 142 ns [6–8]. Therefore, events corresponding to direct annihilation and decay of para-positronium can be reduced to a negligible level by requiring the time difference between de-excitation photon and annihilation photons detection to be larger than e.g. 20 ns. However, such lifetime criterion cannot discriminate pick-off and conversion processes of o-Ps which may lead to the annihilation into $2\gamma$ quanta.

**Fig. 14:**
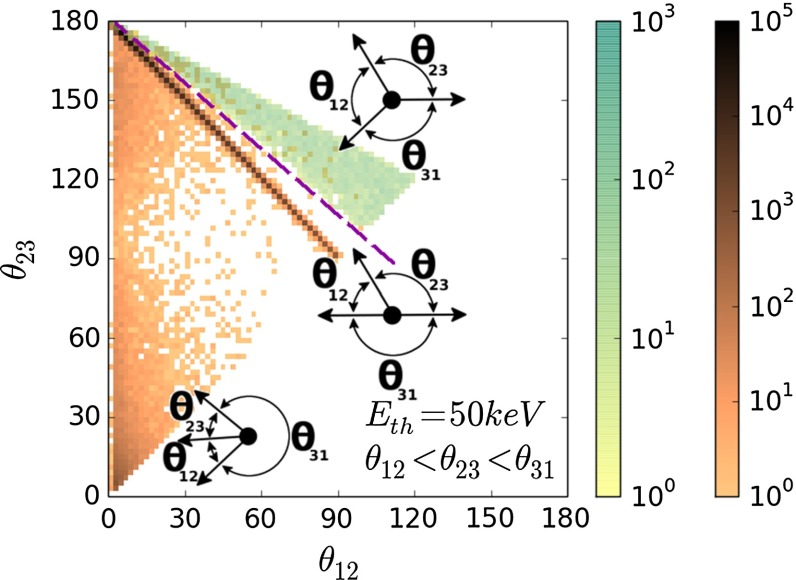
Distribution of $\text{ o-Ps }\rightarrow 3\gamma$ (*green*) and scattered events (*brown*) as a function of $\theta _{12}$ vs $\theta _{23}$ angles. Events, where one of the gamma from $e^+e^- \rightarrow 2 \gamma$ annihilation is registered in the detector while the other is scattered and cause signals in two detectors, lies on the diagonal of the plot. Events where one gamma is missing detection, and the other undergoes two scatterings are localized below the diagonal line. Example of analysis cut, rejecting $3~\%$ of signal and reducing background by factor $10^{4}$, is shown as a *dashed purple line*. Distribution includes the angular resolution of the J-PET detector

Annihilation into $2\gamma$ may mimic a registration of $3\gamma$ annihilation due to the secondary scatterings in the detector. Such scattering is shown pictorially in Fig. 13. For the reduction of this background the following complementary methods can be considered, based on information of:

- relation between position of the individual detectors and the time difference between registered hits,
- angular correlation of relative angles between the gamma quanta propagation directions,
- the distance between the origin of the annihilation (position of the annihilation chamber) and the decay plane.

In Fig. 14 we show as an example spectra the $\theta _{23}$ vs $\theta _{12}$ distribution, where $\theta _{ij}$ are the ordered opening angles ($\theta _{12}< \theta _{23} < \theta _{13}$) between registered gammas. For the $\text{ o-Ps }\rightarrow 3\gamma$ process, due to the momentum conservation, $\theta _{23} > 180^{\circ } - \theta _{12}$ and therefore events corresponding to the $\text{ o-Ps }\rightarrow 3\gamma$ decay will lie above the diagonal, as shown in green colour in Fig. 14. Background events will correspond to points at the diagonal ($\theta _{23} = 180^{\circ } - \theta _{12}$) and below diagonal ($\theta _{23} < 180^{\circ } -\theta _{12}$) as can be inferred from the middle and left panel of Fig. 13. Therefore, one of the possible selection cuts can be applied on ordered opening angles ($\theta _{12}< \theta _{23} < \theta _{13}$) between registered gammas, and is resulting in a decrease of background by a factor $10^{4}$ while, rejecting only $3~\%$ of signal events (see Fig. 14). Combining aforementioned criterion with requirement that registered time difference ($\Delta t$) as a function of detector number ($\Delta ID$) is small ($\Delta t < 0.3$ ns), allows for total reduction of the instrumental background by a factor of $10^{9}$. However, we have to take into account that the remaining background is caused not only by misidentified $2\gamma$ events, but also by true annihilations into $3\gamma$ which may originate from the interaction of the positronium with surrounding electrons and hence will constitute a background for studies of discrete symmetries. Interaction of ortho-positronium with matter is classified into: pick-off annihilations and ortho-para spin conversion. Contribution from these processes depends on the used target material, e.g. in aerogel IC3100 and amberlite porous polymer XAD-4 about 7 and 36 % of ortho-positronium undergo through it, respectively [26]. The events originating from the true of $\text{ o-Ps }\rightarrow 3\gamma$ annihilation process ($N_{o-Ps}$) can be misidentified with the events from the following processes: pick-off process with direct annihilation to $3\gamma$ ($N_{3\gamma \ pick{\text {-}}off}$); pick-off process with annihilation to $2\gamma$ misidentified as $3\gamma$ due to secondary scatterings ($N_{2 \gamma \ pick{\text {-}}off}$); conversion of ortho-positronium to para-positronium with subsequent C symmetry violating decay to $3\gamma$ ($N_{3\gamma \ conv}$); conversion of ortho-positronium to para-positronium with subsequent annihilation to $2\gamma$ misidentified as $3\gamma$ due to the secondary scatterings ($N_{2\gamma \ conv}$).

The conservative upper limit of these background contributions may be estimated as:

6 $$\begin{aligned}&N_{2\gamma \ conv} / N_{o-Ps}< N_{2\gamma \ pick{\text {-}}off} / N_{o-Ps} \nonumber \\&\quad<N_{3\gamma \ conv} / N_{o-Ps} < N_{3\gamma \ pick{\text {-}}off} / N_{o-Ps}, \end{aligned}$$

where:

- $$N_{3\gamma \ pick{\text {-}}off} / N_{o-Ps}< (1-\frac{\tau _{matter}}{\tau _{vacuum}}) / 370 \approx 2\cdot 10^{-4} (\text{ IC3100 }) < 10^{-3} (\text{ XAD-4 })$$;
- $$N_{2\gamma \ pick{\text {-}}off} / N_{o-Ps}< 0.07 \cdot 10^{-9} (\text{ IC3100 }) < 0.36\cdot 10^{-9} (\text{ XAD-4 })$$;
- $$N_{3\gamma \ conv} / N_{o-Ps}< 0.07 \times 2.8 \cdot 10^{-6} (\text{ IC3100 }) <0.36 \times 2.8 \cdot 10^{-6} (\text{ XAD-4 })$$;
- $$N_{2\gamma \ conv} / N_{o-Ps}< 0.07 \cdot 10^{-9} (\text{ IC3100 }) < 0.36\cdot 10^{-9} (\text{ XAD-4 })$$.

In the above estimations the factor $10^{-9}$ denotes the reduction power of the $2\gamma$ events and $2.8\times 10^{-6}$ stands for the upper limit of the C symmetry violation via the $\text{ p-Ps }\rightarrow 3\gamma$ process [39]. The precise control of these contributions will be provided by the measurement of the true $2\gamma$ events with high statistics.

## J-PET performance in $\text{ o-Ps }\rightarrow 3\gamma$ decay measurements

In order to determine the angular and energy resolution we have performed simulations of “point-like” $^{22}$Na source surrounded by water and localized in the geometrical center of the J-PET detector. The conducted simulations accounted for positron emission and thermalisation in the target material, angular and energy distributions of gamma quanta originating from ortho-positronium annihilation and Compton interactions of emitted gamma quanta in the J-PET detector. Details were presented in the Sect. 2. In the next step, based on the simulated data, we reconstructed hit-time and hit-position of the registered gamma quantum interaction in the detector, taking into account the experimentally determined resolutions. Based on obtained informations the reconstruction of angles between gamma quanta and of their energies is performed, as described in the next paragraph.

### Angular and energy resolution

Incident gamma quantum transmits energy as well as momentum to an electron in the plastic scintillator via Compton effect. Due to that, registered signals at the end of the scintillator strips cannot give information about the energy of the incident gamma quantum on the event-by-event basis. However, registration of three gamma quanta hit-position from $\text{ o-Ps }\rightarrow 3\gamma$ annihilation allows reconstruction of their energies based on the energy and momentum conservation.

In CM frame, energies of three gamma quanta from an ortho-positronium annihilation, can be expressed as a functions of angles ($\theta _{12}, \theta _{23}, \theta _{13}$) between momentum vectors (see also Fig. 8, right panel), as follows:

7 $$\begin{aligned} E_1&= - 2m_e\frac{- \cos \theta _{13} + \cos \theta _{12} \cos \theta _{23}}{(-1 + \cos \theta _{12}) (1 + \cos \theta _{12} - \cos \theta _{13} - \cos \theta _{23})}, \nonumber \\ E_2&= - 2m_e \frac{ \cos \theta _{12} \cos \theta _{13} - \cos \theta _{23}}{(-1 + \cos \theta _{12}) (1 + \cos \theta _{12} - \cos \theta _{13} - \cos \theta _{23})}, \nonumber \\ E_3&= 2 m_e \frac{1 + \cos \theta _{12}}{1 + \cos \theta _{12} - \cos \theta _{13} - \cos \theta _{23}}. \end{aligned}$$

The measured positions of gamma interaction in the detector, together with known or reconstructed position of annihilation, allow for $E_i$ determination. The determination of angles requires reconstruction of interaction points and annihilation position. As regards annihilation position we may distinguish two cases, discussed in the next paragraphs.

#### Point-like positronium source

In some cases of discrete symmetries studies positronium will be produced in the well localized material surrounding the “point-like” positron source [1]. Assuming that $\beta ^+$ emitter position corresponds to the ortho-positronium annihilation point, the angles ($\theta _{12}$, $\theta _{13}$ and $\theta _{23}$) between gamma quanta can be determined from registered gamma quanta interaction points ($\mathbf {r}_{hit}$) in the detector. Coordinates *x* and *y* are determined as the centre of the scintillator strip, and therefore the precision of their determination correspond to the geometrical cross section of the scintillator strip. The *z* coordinate is determined from signals arrival time to photomultipliers at the ends of scintillator strip, and its uncertainty is equal to about $\sigma (z) = 0.94$ cm [22, 23]. Uncertainty of $\sigma (\mathbf {r}_{hit})$ determination gives the main contribution to estimation of angular and energy resolutions. The second order effect is an uncertainty originating from non zero boost and distance traveled by positron in matter.

**Fig. 15:**
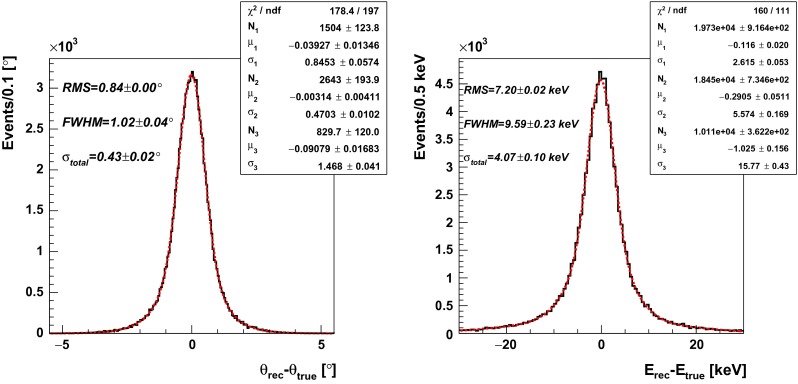
Resulting angular (*left*) and energy (*right*) resolution spectra for “point-like” positronium source with known location and assumed detector resolution $\sigma (T^0_{hit}) = 80$ ps

**Fig. 16:**
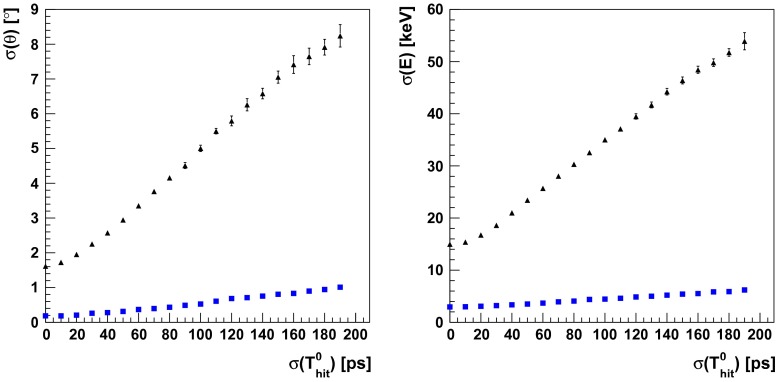
Angular (*left*) and energy (*right*) resolution for the registration of the gamma quanta originating from ortho-positronium annihilation as a function of detector time resolution for “point-like” (*blue box*) and extended (*black triangle*) positronium source

#### Spatially extended positronium source

The angles ($\theta _{12}$, $\theta _{23}$, $\theta _{13}$) and hence a full kinematics of $\text{ o-Ps }\rightarrow 3\gamma$ decay can be also reconstructed in the case of the extended positronium target. For example a target of a cylindrical shape with the diameter of 20 cm was proposed for the production of a linearly polarized positronium [1]. Polarisation can be determined provided that positron emission and positronium formation (approximately the same as annihilation) position are known.

A new reconstruction algorithm that allows reconstruction of ortho-positronium annihilation position for an event by event basis was recently reported [9, 18]. The method based on trilateration allows for a simultaneous reconstruction of both location and time of the annihilation based on time and interaction position of gamma quanta in the J-PET detector. The reconstruction performance strongly depends on detector time resolution ($\sigma (T_{hit})$). Using aforementioned reconstruction algorithm, current J-PET spatial resolution for annihilation point reconstruction is at the level of 1.5 cm along the main detector axis and 2 cm in the transverse plane [18].

#### Performance studies

The angular and energy resolutions for the registration of the gamma quanta from the $\text{ o-Ps }\rightarrow 3\gamma$ decay are established from simulations i.e. the distributions of the differences between generated and reconstructed values of angles and energies. Figure 15 show results obtained under assumption that the hit-time resolution is given by Eqs. 4 and 5. In order to determine the angular and energy resolution the triple Gaussian model, which effectively describes obtained distributions, was applied:

8 $$\begin{aligned} f(x) = \sum _{i=1}^{3} \frac{N_i}{\sqrt{2\pi } \sigma _i} \cdot e^{-\frac{1}{2} \left( \frac{x-\mu _i}{\sigma _i}\right) ^2}, \end{aligned}$$

where $N_i$, $\mu _i$ and $\sigma _i$ were varied in the fit. The total uncertainty was obtained as a standard deviation of the total distribution equivalent to:

9 $$\begin{aligned} \sigma _{total} = \sqrt{ \sum _{i=1}^{3} \left[ \left( \frac{N_i}{\sum _{j=1}^{3} N_j}\right) \cdot \sigma _{i} \right] ^2}. \end{aligned}$$

**Fig. 17:**
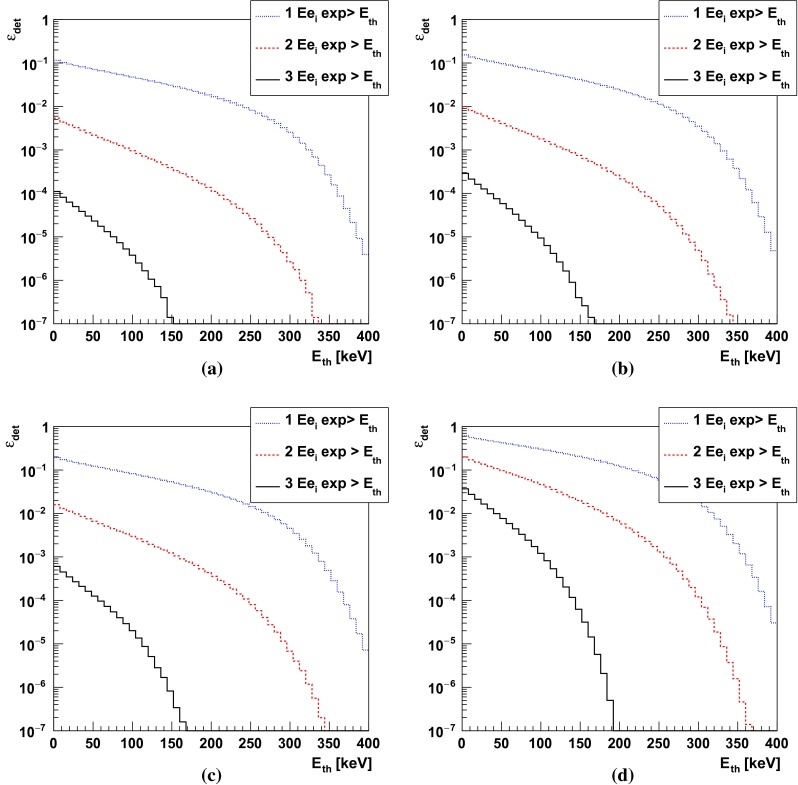
$\text{ oPs } \rightarrow 3 \gamma$ registration efficiency (determined taking into account geometrical acceptance, probability of gamma quanta registration in the plastic scintillator and J-PET detector resolution) as a function of applied threshold for different types of simulated geometries. The shown *dotted*, *dashed* and *solid lines* indicate efficiency assuming that at least one, two or three photons deposited energy above the threshold, respectively

Since the angular and energy resolution strongly depend on hit-time resolution registered in the J-PET detector, the studies of resolution were made for $\sigma (T_{hit}^0)$ in the range from 0 ps to 190 ps. Comparison between obtained resolutions for the “point-like” and extended positronium source is shown in Fig. 16. In both cases energy and angular resolutions are improving with decreasing $\sigma (T_{hit}^0)$, and for presently achieved time resolution of $\sigma (T^0_{hit})$, and well a localized “point-like” positronium source, they amount to $\sigma (\theta ) = {0.4^{\circ }}$ and $\sigma (E_{hit}) = 4.1\,{\mathrm{keV}}$, respectively. In case of the extended positronium source, when the reconstruction of the annihilation point is needed both resolutions increases to $\sigma (\theta ) = {4.2^{\circ }}$ and $\sigma (E_{hit}) = 30\,{\mathrm{keV}}$, respectively.

### J-PET efficiency studies with Monte Carlo simulations

The rate of registered $\text{ o-Ps }\rightarrow 3\gamma$ events in general can be expressed by the formula:

10 $$\begin{aligned} R_{oPs\rightarrow 3\gamma } = A \cdot f_{oPs\rightarrow 3\gamma } \cdot \epsilon _{det}(th) \cdot \epsilon _{ana}, \end{aligned}$$

where *A* is the total annihilation rate (fast timing of applied plastic scintillators allows for usage of the 10 MBq positron source), $f_{oPs\rightarrow 3\gamma }$ is the fraction of annihilations via $\text{ o-Ps }\rightarrow 3\gamma$ process in the target material, $\epsilon _{det}(th)$ is the detector efficiency as a function of applied detection threshold while $\epsilon _{ana}$ denotes selection efficiency used to discriminate between $3\gamma$ and $2\gamma$ events.

The $\epsilon _{det}$ efficiency of the $\text{ o-Ps }\rightarrow 3\gamma$ reconstruction will depend on the energy deposition threshold used in the analysis (see Fig. 12). The hardware threshold at the order of 10 keV [28] will be set to discriminate the experimental noise and later on we will apply further selection threshold based on the measured energy deposition. The probability of registration of 1, 2 or 3 gamma quanta originating from $\text{ o-Ps }\rightarrow 3\gamma$ annihilation ($\epsilon _{det}$) as a function of applied selection threshold in different geometries is shown in Fig. 17. Efficiency $\epsilon _{det}$ contains contribution from geometrical acceptance, probabilities of gamma quanta interaction in applied plastic scintillators and it was determined taking into account the J-PET detector resolution. In our evaluation we assume conservatively that the event selection threshold will be set to 50 keV. A fraction of annihilations via $\text{ o-Ps }\rightarrow 3\gamma$ process is estimated taking into account only longest lived component in two selected materials IC3100 ($f_{oPs \rightarrow 3\gamma } = 16.6~\%$) and XAD-4 ($f_{oPs \rightarrow 3\gamma } =28.6~\%$) [26]. The expected rate of registered signal events is shown in Table 3. Using in the experiment amberlite porous polymer XAD-4 instead of aerogel IC3100 as target material, allows to collect the required statistics almost twice faster, however, resulting with higher systematic uncertainties due to the interaction of positronium with the target material, as discussed in Sect. 4.3.

**Table 3:** Expected rate of registered signal events in different geometries and target materials assuming $10^6$ annihilations per second and requiring energy deposition above 50 keV for all three gamma quanta from $\text{ o-Ps }\rightarrow 3\gamma$ decay

Target material	Rate of registered $\text{ o-Ps }\rightarrow 3\gamma$ events $(\mathrm{s}^{-1})$			
	J-PET	J-PET+1	J-PET+2	J-PET-full
IC3100	15	70	130	10600
XAD-4	25	115	230	18300

## Conclusions

We presented results of Monte Carlo simulations showing that the Jagiellonian-PET multipurpose detector constructed at the Jagiellonian University allows exclusive registration of the decays of ortho-positronium into three photons (o-Ps $\rightarrow 3 \gamma$) providing angular and energy resolution of $\sigma (\theta ) \approx {0.4^{\circ }}$ and $\sigma (E) \approx 4.1\,{\mathrm{keV}}$, respectively.

The achieved results indicate that the J-PET detector gives a realistic chance to improve the best present limits established for the CP and CPT symmetry violations in the decays of positronium [3, 4] by more than an order of magnitude. This can be achieved by (1) collecting at least two orders of magnitude higher statistics, due to the possibility of using a $\beta ^+$ source with higher rate (10 MBq at J-PET vs 0.37 MBq at Gammasphere [3] or 1 MBq at Tokyo University experiment [4]), (2) the enhanced fraction of $3\gamma$ events by the use of the amberlite polymer XAD-4, (3) a measurements with a few times improved angular resolution and (4) about two times higher degree of o-Ps polarization, as shown recently in reference [18]. The limitation on the source activity can be overcome by the J-PET due to the application of plastic scintillators that are characterized by about two orders of magnitude shorter duration of signals, thus decreasing significantly the pile-ups problems with respect to the crystal based detector systems. In addition, the improved angular resolution combined with the superior timing of the J-PET detector (by more than order of magnitude improved with respect to the crystal detectors) and with the possibility of the triggerless registrations [11, 12] of all kind of events with no hardware coincidence window allow suppression and monitoring of the background, due to misidentification of $2\gamma$ events and possible contribution from $3\gamma$ pick-off annihilations.
